# The Prevalence and Genetic Diversity of Avian Malaria in Wild Birds in the Republic of Korea

**DOI:** 10.3390/ani15070957

**Published:** 2025-03-27

**Authors:** Myeongsu Kim, Zun Zun Wut Hmohn, Jae-Ik Han

**Affiliations:** 1 Laboratory of Wildlife Medicine, College of Veterinary Medicine, Jeonbuk National University, Iksan 54596, Republic of Korea; wildvetkim@naver.com (M.K.); zunzunwuthmon@gmail.com (Z.Z.W.H.); 2 Jeonbuk Wildlife Center, Jeonbuk National University, Iksan 54596, Republic of Korea

**Keywords:** avian malaria, *Plasmodium circumflexum*, *Plasmodium relictum*, *Plasmodium homonucleophilum*, TURDUS1

## Abstract

With avian malaria threatening populations of wild birds, this study was conducted to determine its prevalence among wild birds rescued in Jeonbuk state in the Republic of Korea. A total of 1043 wild birds were included in this study from 2017 to 2022. Both quantitative polymerase chain reaction and conventional polymerase chain reaction were used. *Plasmodium* spp. were detected in 75 of the 1043 (7.19%) wild birds. *Plasmodium* spp. were detected every year, and their prevalence was significantly higher in winter and winter migratory birds (*p* < 0.05). The *Plasmodium* spp. detected in wild birds were confirmed to have 30 different lineages. The most frequently detected species was *Plasmodium circumflexum* (cytochrome *b* lineage SW5 and TURDUS1), which is known to prefer cold. In this study, the *Plasmodium* spp. detected in the Republic of Korea were found to contain a high proportion of species preferring colder climates. This suggested that the monitoring of *Plasmodium* spp. in the Republic of Korea should be conducted year round, with a focus on monitoring migratory birds coming from colder climates. Ongoing monitoring and research are needed to understand the impact of *Plasmodium* spp. on wild bird populations.

## Simple Summary

Avian malaria is a well-known vector-borne disease that poses a threat to wild birds, but related research in the Republic of Korea is still limited. This study aimed to assess the prevalence of avian malaria in wild birds rescued in Jeonbuk state, Republic of Korea, including 1043 birds from 2017 to 2022. *Plasmodium* spp. were detected in 7.19% of birds, with higher rates observed in winter and winter migratory birds. A total of 30 *Plasmodium* lineages were identified, with *Plasmodium circumflexum* being the most common. It is known to prefer cold climates, and indeed, it was most commonly found in winter migratory birds. This indicated that the continuous monitoring of avian malaria is necessary even during cold seasons when vectors are less active. The analysis of *Plasmodium* lineages in wild birds in the Republic of Korea was reported for the first time in this study, offering a basis for further studies on emerging *Plasmodium* spp., primary host species, and vectors, as well as highlighting the need for their continued monitoring.

## 1. Introduction

The majority of emerging infectious diseases are associated with wildlife, and their prevalence is gradually increasing [1]. There are various reasons for the increase in wildlife-associated diseases and their transmission, but one of the main factors is the increase in vector-borne diseases due to climate change. Vector-borne diseases refer to diseases transmitted by blood-feeding arthropods such as mosquitoes, ticks, and fleas. Due to climate change, the survival periods of these vectors and the number of regions in which they occur are increasing, leading to an increase in vector-borne diseases [2,3].

Avian malaria is a noteworthy vector-borne disease. It is commonly asymptomatic in healthy birds. It is also known to reduce the population size of endangered species and native birds when adapting to new hosts or being introduced to new areas [4]. Research on the pathogenicity of *Plasmodium* infection in wild birds has been limited. In one study, experimentally infected birds showed sudden death, as well as splenomegaly, hepatomegaly, and cardiomegaly through postmortem examinations [5]. In another study, it was found that birds infected with *Plasmodium* spp. have significantly shorter telomeres, which is associated with a shorter lifespan, compared to those without infections, indicating a significant pathogenicity in wildlife [6]. The transmission of *Plasmodium* spp. can be accelerated by migrant birds, and illegal trade also contributes to their spread. *Plasmodium* infections not only worsen the condition of infected birds but also facilitate its transmission to the wild, impacting the ecosystem of wild avifauna [7,8]. Monitoring vector-borne diseases is important not only for controlling the spread of disease but also for the conservation of ecosystems, including wild birds.

Despite the importance and risk of avian malaria, few studies have been conducted in the Republic of Korea. There was a reported case of a captive Magellan penguin (*Spheniscus magellanicus*) infected with *Plasmodium* spp. in the Republic of Korea [9]. In other studies, following data analysis, it appeared that with each passing year, there was an increase in the diversity of *Plasmodium* spp. detected, as well as their prevalence [10,11]. This underscores the necessity for research into the occurrence and patterns of avian malaria in the Republic of Korea.

This study aimed to analyze the prevalence and genetic diversity of avian malaria among wild birds rescued in Jeonbuk state from 2017 to 2022. Through this analysis, we aimed to investigate the prevalence and genetic diversity of avian malaria transmission in the Republic of Korea.

## 2. Materials and Methods

### 2.1. Sample Collection

**Figure 1:**
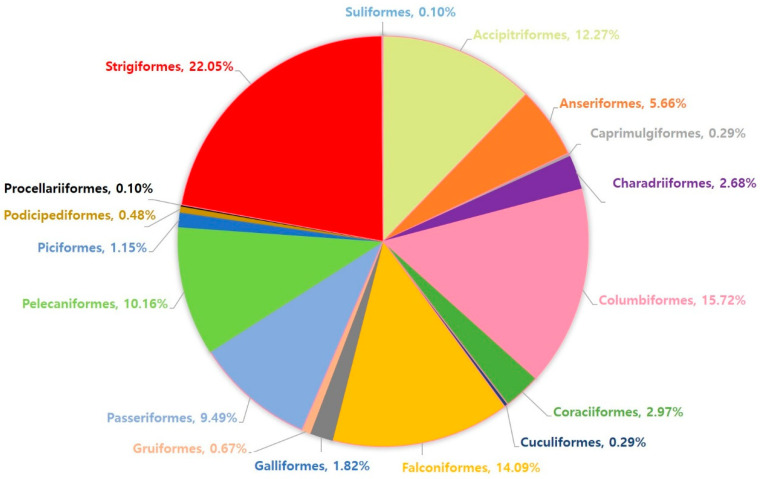
The proportions of the orders of wild birds examined in this study.

A total of 1043 wild birds rescued between January 2017 and December 2022 were included in this study (Supplementary Table S1). The birds examined belonged to 17 orders, 29 families, and 82 species. The order Strigiformes was the most abundant (230/1043; 22.05%), followed by Columbiformes (164/1043; 15.72%; Figure 1), in this study.

The prevalence of *Plasmodium* spp. was examined based on not only the species and genus of birds but also their seasonal migration, divided into four groups: resident birds (birds that stay in one place throughout the year), summer migratory birds (birds that migrate to a region during the summer), winter migratory birds (birds that migrate to a region during the winter), and passage migrant bird (birds that stop over temporarily during their migration between breeding and wintering grounds) [12].

All blood samples were either collected and used during medical procedures or collected after death during a necropsy to determine the cause of death. After testing for treatment, the remaining samples were stored at −20 °C and thawed before use. Information on the examined patients (rescued terrain and sampling month) was collected retrospectively.

### 2.2. DNA Extraction

The blood samples stored in EDTA were thawed; then, DNA extraction was performed using a QIAamp^®^ DNA Mini Kit (Qiagen, Hilden, Germany) according to the manufacturer’s instructions. The extracted nucleic acids were stored at −20 °C until the next procedure.

### 2.3. Real-Time Polymerase Chain Reaction

A real-time PCR was conducted to detect *Plasmodium* spp. quickly and with high sensitivity, as described previously (Table 1) [13]. PCR amplification was performed in a total volume of 20 µL, including TaqMan^®^ Fast Advanced Master Mix (Thermo Fisher Scientific, Waltham, MA, USA), 0.2 uM of each primer, and 0.1 uM of probe. The thermal cycler protocol consisted of incubation at 50 °C for 2 min and 95 °C for 20 s, as well as 40 cycles at 95 °C for 1 s and at 60 °C for 20 s. Only samples with a cycle threshold (Ct) of 35 or less were considered positive and utilized in the next step. All procedures included previously obtained *Plasmodium*-positive samples as positive controls and distilled water as negative controls.

### 2.4. Conventional Polymerase Chain Reaction

Conventional PCR, with primers 3760F and 4292Rw2 for the cytochrome *b* gene, as described previously, was performed to obtain sequences and conduct a phylogenetic analysis (Table 2) [14,15]. If the fragment was not amplified, a follow-up reaction was performed with smaller fragments using F1 and 4292Rw2. The PCR amplicons had an expected size of 533 and 433 base pairs, respectively. PCR amplification was performed in a total volume of 50 µL, including the HotstarTaq Master Mix Kit (Qiagen), 0.2 µM of each primer, and a template volume of 2 µL. The mixture was denatured for 15 min at 95 °C, followed by 40 cycles at 95 °C for 45 s, 51 °C for 45 s, and 72 °C for 1 min, before finally being extended at 72 °C for 7 min and then maintained at 4 °C. The positive control and negative control were used in the same way as in the real-time polymerase chain reaction. If positive, the PCR products were bi-directionally sequenced. After this, the sequences were assembled and then subsequently aligned with a known *Plasmodium* spp. genomic sequence using the Basic Local Alignment Search Tool (BLAST, https://blast.ncbi.nlm.nih.gov/Blast.cgi accessed on 24 September 2024) in GenBank and in the MalAvi database to confirm their identification.

### 2.5. Climate Data: Temperature and Precipitation

The seasons were categorized according to the weather in the Republic of Korea as follows: spring (March to May), summer (June to August), autumn (September to November), and winter (December to February). During the research period, the average monthly temperature and precipitation for each year were obtained from the Republic of Korea Meteorological Administration website [16] to analyze their correlation with the prevalence of *Plasmodium* spp.

### 2.6. Lineage Analysis

All sequences were aligned using ClustalX version 1.8 [17]. The identified sequences were aligned with sequences from the MalAvi database to determine whether they represented new lineage sequences [14]. These sequences were selected from the MalAvi database due to a similarity of over 98% with the sequences of *Plasmodium* lineages obtained in this study. The diversity of the lineages was confirmed using the Shannon diversity index (H’) for each year. All the sequences detected in this study were deposited in the NCBI GenBank database (accession numbers PP500635 to PP500709).

### 2.7. Phylogenetic Analysis

Median-joining phylogenies were generated using PopART version 1.7 [18]. To understand the within-species variation in *Plasmodium* spp. and their genetic diversity, two networks were constructed as follows: (1) the lineages were classified according to their detection in hosts by avian order; and (2) the lineages were classified according to the rescue regions of their hosts. Only sequences obtained in this study were used.

### 2.8. Statistical Analysis

Statistical analysis was performed using the statistical software SPSS 20 (IBM SPSS Statistics, Chicago, IL, USA). To assess the effects of independent variables on *Plasmodium* spp. prevalence, the Chi-square test (year, order of host, species of host, seasonal movement of host, and seasons) and bivariate correlations (temperature and precipitation) were used. A value of *p* < 0.05 was considered statistically significant.

## 3. Results

### 3.1. Prevalence of Plasmodium spp.

Out of 1043 wild birds, 75 tested positive (75/1043, 7.19%). Their annual prevalence values were as follows: 9.48% (11/116) in 2017, 6.83% (14/205) in 2018, 8.81% (17/193) in 2019, 7.04% (10/142) in 2020, 4.44% (10/225) in 2021, and 8.02% (13/162) in 2022 (Supplementary Table S2). There was no significant difference in prevalence across years (*p* = 0.49).

*Plasmodium* spp. were detected in wild birds belonging to 32 species across 13 families and 9 orders. Their prevalence by order was significantly highest in Passeriformes (24/99; 24.24%), Galliformes (3/19; 15.79%), and Anseriformes (9/59; 15.25%), with 10 or more individuals tested in each species (Supplementary Table S3; *p* < 0.05). Their prevalence by species was significantly highest in Eurasian magpies (*Pica pica*) (9/53; 16.98%) and common buzzards (*Buteo buteo*) (7/43; 16.28%), with 30 or more individuals tested in each species (Supplementary Table S4; *p* < 0.05).

*Plasmodium*’s prevalence according to the seasonal movement of birds was ranked as follows: winter migratory birds (25/191; 13.09%), passage migrant birds (1/8; 12.50%), summer migratory birds (19/289; 6.57%), and resident birds (30/555; 5.41%) (Supplementary Table S5). The seasonal movement of the host was found to have a significant impact on prevalence (*p* < 0.05). Prevalence by season was highest in winter (27/225; 12.00%), followed by autumn (23/239; 9.66%), spring (13/250; 5.20%), and summer (12/329; 3.65%) (Supplementary Table S6). The seasons also showed a significant difference, with notably higher rates during winter and significantly lower rates during summer (χ^2^(3) = 13.11; *p* = 0.004). Supplementary Table S7 contains information about *Plasmodium*-positive wild birds.

### 3.2. Monthly Average Temperatures and Precipitation for Each Year

The average temperatures during the winter of 2017, 2018, and 2022 were below freezing. The temperature in November 2022 was approximately twice as high as that in other years. The spring of 2018 and summer of 2020 had a higher precipitation than those of other years (Supplementary Table S8). During the research period, temperature (r = −0.371; *p* = 0.001) and precipitation (r = −0.275; *p* = 0.019) showed weak negative correlations with the prevalence of *Plasmodium* spp.

### 3.3. Lineage

**Figure 2:**
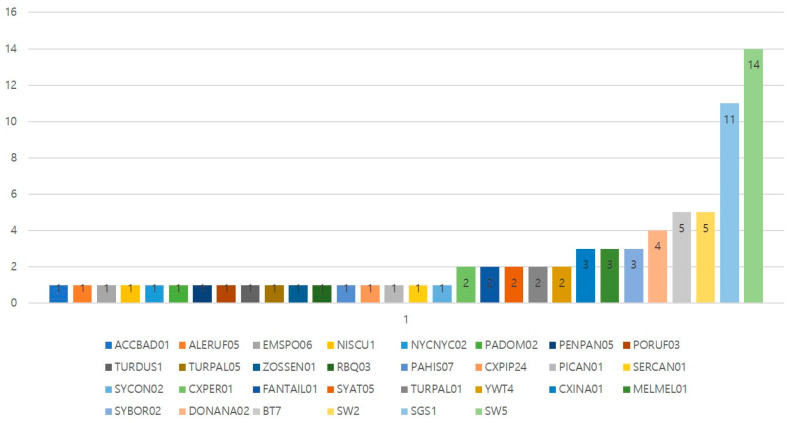
*Plasmodium* spp. lineage diversity in relation to the total number of detected lineages.

In total, 30 different *Plasmodium* lineages were found in 75 birds (Supplementary Table S9). SW5, identified as *Plasmodium circumflexum*, was the most detected (14/75; 18.67%), followed by SGS1, identified as *Plasmodium relictum* (11/75; 14.67%) (Figure 2). The Shannon diversity indexes were as follows: 2017 (H’ = 1.29), 2018 (H’ = 1.59), 2019 (H’ = 2.23), 2020 (H’ = 2.03), 2021 (H’ = 2.30), and 2022 (H’ = 2.10). The year 2021 showed the highest diversity of detected lineages, with all 10 *Plasmodium* spp. representing different lineages. In contrast, 2017 had the lowest diversity, with only 4 out of 11 *Plasmodium* spp. representing different lineages. Among the bird species detected two or more times, the only bird species that showed the same lineage was the long-eared owl, while the other bird species either exhibited different lineages in different years or belonged to different lineages even if detected in the same year.

Upon analyzing the diversity of lineages by order (Supplementary Table S9), it was observed that the *Plasmodium* lineages from Columbiformes, Galliformes, and Gruiformes were all different. In Accipitriformes, BT7 had the highest prevalence (4/12; 33.33%). In Anseriformes, SW5 had the highest prevalence (5/9; 55.56%), while in Strigiformes, SW5 had the highest prevalence (3/6; 50.00%). In Passeriformes, SGS1 had the highest prevalence (10/24; 41.67%). The lineages from other orders had similar proportions. Regarding the diversity of the *Plasmodium* lineages detected in each terrain (water, urban, farmland, and forest), based on rescue area, it was noticeable that there was not a particularly high number of any lineage detected in each terrain.

### 3.4. Median-Joining Network

**Figure 3:**
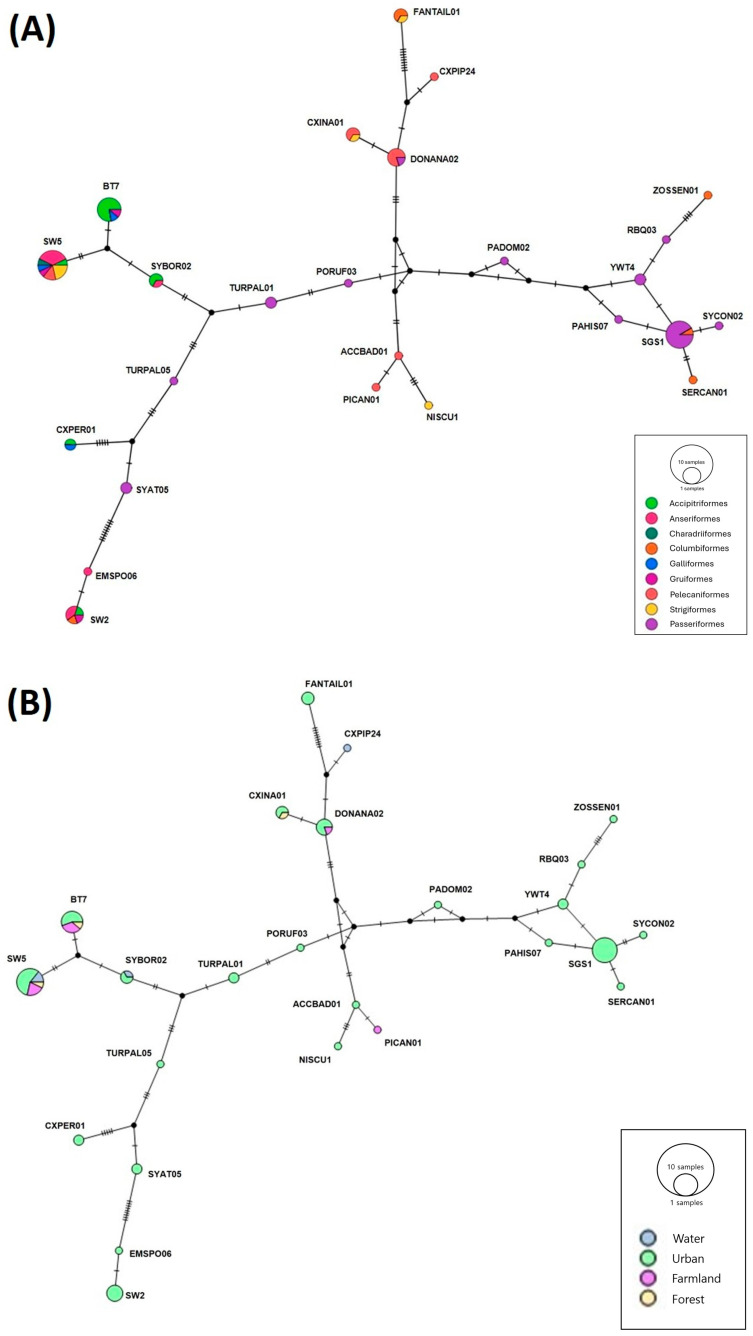
Median-joining network of *Plasmodium* lineage by avian order (**A**) and rescued terrain (**B**) (epsilon = 0). Circles denote lineage, and their sizes are proportional to lineage frequencies. Colors denote host order (**A**) and rescue terrain (**B**). Missing lineages are indicated by small black circles, and hash marks represent mutation steps between lineages.

The lineages of *Plasmodium* spp. were classified according to the order of their hosts (Figure 3A) and rescue regions (Figure 3B). Both networks were similarly constructed. The evolutionary differences among the *Plasmodium* lineages detected in Passeriformes appeared to be the most diverse, with a significant number of lineages exclusively detected in Passeriformes. In terms of rescue regions, *Plasmodium* spp. appeared to be the most diverse in urban ones. SW5 was distributed most broadly in terms of both host and geography.

## 4. Discussion

In this study, we investigated the prevalence and genetic diversity of avian malaria in wild birds in Jeonbuk state from 2017 to 2022. Upon comparing its prevalence by year, the results showed that the highest level was observed in 2017, reaching 9.48%. In contrast, the prevalence was lowest in 2021 at 4.44%. There were variations in prevalence across years, and no consistent pattern was evident. In the winters of 2017, 2018, and 2022, which were colder compared to the other years, there was a higher prevalence of *Plasmodium* spp. In the spring of 2017 and 2018, as well as the summer of 2020, although there was not much of a difference in temperature compared to the other years, *Plasmodium* sp. was never detected. It is generally known that climate change tends to increase vector populations. The presence of vectors is likely the most closely related factor to the occurrence of avian malaria. However, various factors, such as host species or habitats, also influence the prevalence of malaria [19]. Therefore, it seems difficult to predict fluctuations in prevalence by year, and confirming a consistent pattern appears challenging. Despite the variation in *Plasmodium*’s prevalence across years, the consistent detection of avian malaria in Jeonbuk state indicated the presence of sufficient vectors and suitable environmental conditions for disease transmission. Moreover, the fact that avian malaria was consistently detected at a significant rate suggests that it has become an endemic disease in Jeonbuk state.

In terms of the comparison of prevalence by host orders, the highest prevalence was found in the Passeriformes; a high prevalence was also seen in the Anseriformes. Passeriformes are the largest group among bird orders, and research on this order has been relatively more extensive compared to other orders [20]. Previous studies have already confirmed that the prevalence of *Plasmodium* spp. is high in this group and that the parasites they carry are genetically diverse [21,22]. On the other hand, the fact that Anseriformes had a high prevalence was noteworthy. The Anseriformes detected in this study, including the mallard (*Anas platyrhynchos*), gadwall (*Anas strepera*), bean goose (*Anser fabalis*), green-winged teal (*Anas crecca*), spot-billed duck (*Anas poecilorhyncha*), and whooper swan (*Cygnus cygnus*), are all classified as winter migratory birds in the Republic of Korea [23]. This suggested that the high prevalence observed in winter could be related to the high prevalence in winter migratory birds.

The comparison of prevalence by species from 2017 to 2022 revealed that a resident bird, *P. pica*, had the highest prevalence, followed by a winter migratory bird, *B. buteo* (with more than 30 birds tested in each species). Among the 32 species with positive results for *Plasmodium* spp., there were 12 species of resident birds, 10 species of winter migratory birds, 9 species of summer migratory birds, and 1 species of passage migrant bird, indicating that the *Plasmodium* spp. were detected in various resident birds. Given that migratory birds typically play a more significant role in disease transmission, while resident birds are generally considered less important [24], the fact that a greater variety of *Plasmodium* spp. was detected in resident birds, and that these birds had a higher prevalence, was noteworthy. This could serve as another indication that avian malaria is already endemic in Jeonbuk state.

Upon comparing the prevalence of host species according to the seasonal movement of host species from 2017 to 2022, winter migratory birds had the highest prevalence (13.09%), while resident birds had the lowest prevalence (5.42%). Upon comparing winter migratory birds, summer migratory birds, resident birds, and passage migratory birds, winter migratory birds showed a significantly higher prevalence of *Plasmodium* spp. Similarly, when comparing the prevalence by season, winter significantly had the highest rate (12.00%), while summer significantly had the lowest (3.65%). It is commonly known that the transmission of avian malaria typically occurs where vectors thrive, as well as when there is increased contact among hosts or during breeding seasons when there are many young birds [25]. Based on this, the season with the highest prevalence in the Republic of Korea would be from around spring to autumn [26]. The fact that winter and winter migratory birds showed the highest prevalence was noteworthy. In 2022, *Plasmodium* spp. were not detected in summer migratory birds, and there were instances where no detections were recorded even during spring, when there is increased contact among individuals (2017, 2018). Additionally, there were cases where no detections were observed during summer, a season when young individuals are born and vectors are most active (in 2020). Avian malaria is known to primarily occur in tropical and subtropical regions where vectors thrive. Vector-borne diseases are generally considered to increase as the areas preferred by vectors expand due to climate change [27]. In some years of this study, the *Plasmodium* sp. was not detected during the vectors’ preferred seasons of spring and summer. However, in the same year, *Plasmodium* spp. were detected in winter despite the unfavorable conditions for mosquitoes. Additionally, the absence of detections of *Plasmodium* spp. in summer migratory birds, which inhabit environments favorable for vector presence, showed that the prevalence of vector-borne diseases could increase not only due to vectors but also due to other factors such as stress or relapses of the parasite. A weak negative correlation was observed between the temperature and the prevalence of *Plasmodium* spp., as well as between precipitation and the prevalence of *Plasmodium* spp. during the study period (2017–2022). This could also serve as additional evidence that in the Republic of Korea, factors other than the presence of vectors may influence the prevalence of *Plasmodium* infections in wild birds.

It was confirmed that of the *Plasmodium* spp. isolated from the 75 wild birds, 32 species with 30 different lineages of *Plasmodium* spp. were identified. Except for the long-eared owl, there were no instances in which identical lineages were detected in the same host species. This demonstrates the genetic diversity of *Plasmodium* spp. harbored by wild birds, demonstrating variability even within the same species.

*P*. *circumflexum* (cytochrome *b* lineage SW5 and TURDUS1) was the most detected species in this study. *P. circumflexum* (cytochrome *b* lineage SW5 and TURDUS1) was detected in 7 orders, 7 families, and 10 species (Supplementary Table S9). Among the 15 birds in which *P. circumflexum* (cytochrome *b* lineage SW5 and TURDSUS1) was detected, 10 were winter migratory birds. In total, 10 out of the 25 winter migratory birds in which *Plasmodium* spp. was detected in this study were infected with *P. circumflexum* (cytochrome *b* lineage SW5 and TURDSUS1). *P. circumflexum* (cytochrome *b* lineage SW5 and TURDUS1) is known as a newly emerging *Plasmodium* spp. in countries with cold climates [28]. This could serve as one of the reasons for the increased prevalence of the *Plasmodium* spp. detected in winter and winter migratory birds in this study. Lineage SW5 has especially been primarily identified in wild birds from different countries [29]. While lineage SW5 is relatively well known, lineage TURDUS1 remains poorly understood, particularly regarding the vectors that primarily transmit it and the main host species it infects [30]. Research on *Plasmodium* spp. in wild birds has been limited in the Republic of Korea [9,10,11], and an analysis of the *Plasmodium* lineage was conducted for the first time in the Republic of Korea in this study. Therefore, the detection of all lineages in Korean wild birds was significant, but especially in the case of TURDUS1, where the vector is unknown; this study could provide valuable information in identifying it.

*P*. *relictum* (cytochrome *b* lineage SGS1) was the second most detected *Plasmodium* spp. in this study. *P. relictum* is known to be widely distributed worldwide and to show a high prevalence in a variety of hosts [31]. *P. relictum* is also known to be a major species that affects the native bird populations of the region when introduced to new areas [32,33]. In contrast to *P. circumflexum* (cytochrome *b* lineage SW5 and TURDUS1), which was detected in a variety of orders, *P. relictum* (cytochrome *b* lineage SGS1) was only detected in Passeriformes and Columbiformes (Supplementary Table S9). *P. relictum* (cytochrome *b* lineage SGS1) accounted for the highest proportion of *Plasmodium* spp. detected in resident birds (33.3%) in this study. The fact that 33.3% of *Plasmodium* spp. detected in resident birds in the Republic of Korea were related to *P. relictum* (cytochrome *b* lineage SGS1) suggests that this species is a major pathogen of avian malaria in resident birds in the Republic of Korea. *P. relictum* (cytochrome *b* lineage SGS1), which poses a threat to native bird populations in new regions [32,33], is likely to have a relatively low risk to resident birds in the Republic of Korea, where *Plasmodium* spp. were already endemic.

*Plasmodium homonucleophilum* (cytochrome *b* lineage SW2) was also one of the lineages primarily detected in this study (6.66%). Little is known about the host or vector of *P. homonucleophilum* (cytochrome *b* lineage SW2), and studies on its pathogenicity and transmission are ongoing [34,35]. The detection of *P. homonucleophilum* (cytochrome *b* lineage SW2) in five different species in this study could provide valuable evidence for understanding its host species or transmission.

## 5. Conclusions

This study confirmed that *Plasmodium* spp. were already an endemic disease in wild birds in the Republic of Korea, with a higher prevalence in a cold climate, differing from previous research. Despite being a vector-borne disease, *Plasmodium* spp. can be detected year round, and the higher prevalence in winter suggests that monitoring and identifying transmission pathways throughout all seasons is necessary. By identifying the *Plasmodium* lineages detected in wild birds in the Republic of Korea, the species most frequently detected in this study was *P. circumflexum*, which prefers cold climates. Ongoing research is needed to understand how various *Plasmodium* spp. affect native bird populations and how they might be transmitted through migratory birds.

## Figures and Tables

**Table 1:** Information of primer/probe sets for the detection of *Plasmodium* spp. [13].

Name	Sequences (5′ to 3′)	Target Gene	Length of Amplicon (bp)
18sPlasm7	AGC CTG AGA AAT AGC TAC CAC ATC TA	18s rDNA	60
18sPlasm8	TGT TAT TTC TTG TCA CTA CCT CTC TTC TTT		
Plasm Hyb2	FAM-CAG CAG GCG CGT AAA TTA CCC AAT TC-BHQ1		

**Table 2:** Information of primer sets for the detection and sequencing of *Plasmodium* spp. [14,15].

Name	Sequences (5′ to 3′)	Target Gene	Length of Amplicon (bp)
3760F	GAGTGGATGGTGTTTTAGAT	Cytochrome *b*	533
4292Rw2	TGGAACAATATGTARAGGAGT		
F1	CATATTTACCTTTATCATGGAT		433

## Data Availability

All data presented in this study are available from the corresponding authors on request.

## Supplementary Materials

The following supporting information can be downloaded at https://www.mdpi.com/article/10.3390/ani15070957/s1. Table S1: Information on the wild birds included in this study; Table S2: Yearly prevalence of *Plasmodium* spp.; Table S3: Prevalence of *Plasmodium* spp. according to host order; Table S4: Prevalence of *Plasmodium* spp. according to host species; Table S5: Prevalence of *Plasmodium* spp. according to seasonal movement of host species; Table S6: Prevalence of *Plasmodium* spp. according to seasons; Table S7: Information on the wild birds included in this study; Table S8: Monthly average temperatures (°C) and precipitation (mm) for each year; Table S9: Birds with positive results of *Plasmodium* spp. and their lineages.
