# Supplementary material for: The Prevalence and Genetic Diversity of Avian Malaria in Wild Birds in the Republic of Korea
Source: Animals (Basel). 2025 Mar 27;15(7):957. doi: 10.3390/ani15070957 (PMC11987983; doi:10.3390/ani15070957)
Supplement: Supplementary file 1 [file animals-15-00957-s001.zip › animals-3498180-supplementary.pdf]

Table S1. Information on the wild birds included in this study

Order	Family	Common name	Scientific name	L	No
**Accipitriformes**	Accipitridae	Goshawk	*Accipiter gentilis*	W	39
		Cinereous vulture	*Aegypius monachus*	W	16
		Common buzzard	*Buteo buteo*	W	43
		Japanese lesser sparrow hawk	*Accipiter gularis*	S	7
		Osprey	*Pandion haliaetus*	P	2
		Siberian honey buzzard	*Pernis ptilorhynchus*	P	2
		Chinese sparrow hawk	*Accipiter soloensis*	S	2
		Sparrow haw	*Accipiter nisus*	W	15
		Hen harrier	*Circus cyaneus*	W	2
**Anseriformes**	Anatidae	Baikal teal	*Anas formosa*	W	1
		Pintail	*Anas acuta*	W	2
		Tufted duck	*Aythya fuligula*	W	1
		White-fronted goose	*Anser albifrons*	W	3
		Green-winged teal	*Anas crecca*	W	4
		Gadwall	*Anas strepera*	W	2
		Mandarin duck	*Aix galericulata*	R	3
		Mallard	*Anas platyrhynchos*	W	8
		Whooper swan	*Cygnus cygnus*	W	3
		Bean goose	*Anser fabalis*	W	8
		Spot-billed duck	*Anas poecilorhyncha*	R	22
		Pochard	*Aythya ferina*	W	2
**Caprimulgiformes**	Caprimulgidae	Jungle nightjar	*Caprimulgus indicus*	S	3
**Charadriiformes**	Alcidae	Ancient murrelet	*Synthliboramphus antiquus*	W	1
	Laridae	Common gull	*Larus canus*	W	1
		Black-tailed gull	*Larus crassirostris*	R	16
		Slaty-backed gull	*Larus schistisagus*	W	1
	Scolopacidae	Common snipe	*Gallinago gallinago*	P	1
		Woodcock	*Scolopax rusticola*	W	6
		Whimbrel	*Numenius phaeopus*	P	2

**Columbiformes**	Columbidae	Oriental Turtle Dove	*Streptopelia orientalis*	R	65
		Domestic pigeon	*Columba livia domestica*	R	99
**Coraciiformes**	Alcedinidae	Common kingfisher	*Alcedo atthis bengalensis*	S	2
		Ruddy kingfisher	*Halcyon coromanda*	S	2
	Coraciidae	Broad-billed roller	*Eurystomus orientalis*	S	24
	Upupidae	Hoopoe	*Upupa epops saturata*	S	3
**Cuculiformes**	Cuculidae	Oriental cuckoo	*Cuculus saturatus*	S	1
		Common cuckoo	*Cuculus canorus*	S	2
**Falconiformes**	Falconidae	Hobby	*Falco subbuteo*	S	15
		Peregrine Falcon	*Falco peregrinus*	R	5
		Kestrel	*Falco tinnunculus*	R	127
**Galliformes**	Galliformes	Pheasant	*hasianus colchicus*	R	16
		Common quai	*Coturnix japonica*	R	3
**Gruiformes**	Rallidae	Eurasian Coot	*Fulica atra*	R	6
		Moorhen	*Gallinula chloropus*	S	1
**Passeriformes**	Bombycillidae	Japanese waxwing	*Bombycilla japonica*	W	1
	Corvidae	Carrion crow	*Corvus corone orientalis*	R	2
		Eurasian magpie	*Pica pica*	R	53
		Eurasian jay	*Garrulus glandarius*	R	2
		Jungle crow	*Corvus macrorhynchos*	R	2
		Rook	*Corvus frugilegus*	W	2
		Azure-winged magpie	*Cyanopica cyanus*	R	5
	Fringillidae	Oriental greenfinch	*Carduelis sinica ussuriensis*	R	1
	Oriolidae	Black-naped oriole	*Oriolus chinensis*	S	4
	Passeridae	Tree sparrow	*Passer montanus*	R	1
	Pycnonotidae	Brown-eared Bulbul	*Microscelis amaurotis*	R	16
	Sturnidae	Gray starling	*Sturnus cineraceus*	S	2
	Turdidae	Gray-backed Thrush	*Turdus hortulorum*	S	2
		White's ground thrush	*Zoothera dauma*	S	6
**Pelecaniformes**	Ardeidae	Cattle egret	*Bubulcus ibis*	S	19
		Green-backed heron	*Butorides striatus*	S	6
		Chinese little bittern	*Ixobrychus sinensis*	S	4

		Little egret	*Egretta garzetta*	S	12
		Gray heron	*Ardea cinerea*	S	26
		Large egret	*Egretta alba modesta*	S	15
**Pelecaniformes**	Ardeidae	Intermediate egret	*Mesophoyx intermedia*	S	12
		Schrenck's bittern	*Ixobrychus eurhythmus*	S	5
		Black-crowned night heron	*Nycticorax nycticorax*	S	5
**Pelecaniformes**	Threskiornithidae	Black-faced spoonbill	*Platalea minor*	S	1
		Eurasian spoonbill	*Platalea leucorodia*	W	1
**Piciformes**	Picidae	Great spotted woodpecker	*Dendrocopos major*	R	2
		Grey-headed woodpecker	*Picus canus*	R	5
		White-backed woodpecker	*Dendrocopos leucotos*	R	5
**Podicipediformes**	Podicipedidae	Little grebe	*Tachybaputus ruficollis*	R	1
		Great crested grebe	*Podiceps cristatus*	2	4
**Procellariiformes**	Procellariidae	Streaked shearwater	*Calonectris leucomelas*	S	1
**Strigiformes**	Strigidae	Scops owl	*Otus scops*	S	26
		Brown hawk-owl	*Ninox scutulata*	S	82
		Eurasian eagle-owl	*Bubo bubo*	R	84
		Korean wood owl	*Strix aluco*	R	13
		Long-eared owl	*Asio otus*	W	8
		Collared scops owl	*Otus bakkamoena ussuriensis*	W	17
**Suliformes**	Sulidae	Red-footed Booby	*Sula sula*	P	1

L indicates seasonal movement of birds, W, winter migratory birds; S, summer migratory birds; R, resident bird; P, passage migrant bird; No, number of birds

Table S2. Yearly prevalence of *Plasmodium* spp.

**Year**	**No. bird**	**No. positive**	**PR* (%)**	**95% CI ****
2017	116	11	9.48	(4.15, 14.81)
2018	205	14	6.83	(3.38, 10.28)
2019	193	17	8.81	(4.81, 12.81)
2020	142	10	7.04	(2.83, 11.25)

2021	225	10	4.44	(1.75, 7.14)
2022	162	13	8.02	(3.84, 12.21)
Total	1043	75	7.19	(5.62, 8.76)

PR*: prevalence, CI**: confidence interval.

Table S3. Prevalence of *Plasmodium* spp. according to host order

**Order**	**No. bird**	**No. positive**	**PR* (%)**	**95% CI ****
Accipitriformes	128	12	9.38	(4.33, 13.70)
Anseriformes	59	9	15.25	(4.68, 19.94)
Charadriiformes	28	1	3.57	(3.51, 7.08)
Columbiformes	164	6	3.66	(1.47, 5.12)
Galliformes	19	3	15.79	(8.37, 24.15)
Gruiformes	7	3	42.86	(18.70, 61.56)
Passeriformes	99	24	24.24	(4.31, 28.55)
Pelecaniformes	106	11	10.38	(2.96, 13.34)
Strigiformes	230	6	2.61	(1.05, 3.66)

PR*: prevalence, CI**: confidence interval.

Table S4. Prevalence of *Plasmodium* spp. according to host species

**Species**	**No. bird**	**No. positive**	**PR* (%)**	**95% CI ****
Pica pica	53	9	16.98	(6.87, 27.09)
Oriolus chinensis	4	1	25.00	(0, 67.44)
Phasianus colchicus	16	2	12.50	(0, 28.71)
Ixobrychus sinensis	4	3	75.00	(32.56, 100.00)
Corvus frugilegus	2	2	100.00	(0, 100.00)
Turdus hortulorum	2	1	50.00	(0, 100.00)
Buteo buteo	43	7	16.28	(5.24, 27.31)
Coturnix japonica	3	1	33.33	(0, 86.68

Scolopax rusticola	6	1	16.67	(0, 46.49)
Streptopelia orientalis	66	2	3.03	(0, 7.17)
Cyanopica cyanus	5	1	20.00	(0, 55.06)
Fulica atra	6	3	50.00	(9.99, 90.01)
Pernis ptilorhynchus	2	1	50.00	(0, 100.00)
Ninox scutulata	82	2	2.44	(0, 5.78)
Bubo bubo	83	1	1.20	(0, 3.55)
Anas crecca	4	1	25.00	(0, 67.44)
Anas strepera	2	1	50.00	(0, 100.00)
Garrulus glandarius	2	1	50.00	(0, 100.00)
Ardea cinerea	26	1	3.85	(0, 11.24)
Egretta alba modesta	15	2	13.33	(0, 30.54)
Microscelis amaurotis	16	4	25.00	(3.78, 46.22)
Columba livia domestica	100	4	4.00	(0.16, 7.84)
Accipiter gentilis	39	4	10.26	(0.73, 19.78)
Anas platyrhynchos	7	4	57.14	(20.48, 93.80)
Asio otus	9	3	33.33	(2.53, 64.13)
Cygnus cygnus	3	1	33.33	(0, 86.88)
Anser fabalis	8	1	12.50	(0, 35.42)
Ixobrychus eurhythmus	5	4	80.00	(44.94, 100.00)
Corvus macrorhynchos	2	1	50.00	(0, 100.00)
Zoothera dauma	6	4	66.67	(28.95, 100.00)
Bubulcus ibis	17	1	5.88	(0, 17.07)
Anas poecilorhyncha	22	1	4.55	(0, 13.25)

Table S5. Prevalence of *Plasmodium* spp. according to seasonal movement of host species

	**Year**	**2017**	**2018**	**2019**	**2020**	**2021**	**2022**	**Total**
**Seasonal movement**								
**Winter**	**No. birds**	28	49	30	23	33	28	191

**Migratory bird**	**No. positive**	7	5	3	3	1	6	25
	**PR (%)**	25.00	10.20	10.00	13.04	3.03	21.43	13.09
	**95% CI**	(8.96, 41.04)	(1.73, 18.68)	(0, 20.74)	(0, 26.81)	(0, 8.88)	(6.23, 36.63)	(8.31, 17.87)
**Summer migratory bird**	**No. birds**	30	60	52	42	67	38	289
	**No. positive**	3	3	5	3	5	0	19
	**PR (%)**	10.00	5.00	9.62	7.14	7.46	0.00	6.57
	**95% CI**	(0, 20.74)	(0, 10.51)	(1.60, 17.63)	(0, 14.93)	(1.17, 13.76)	-	(3.72, 9.43)
**Resident bird**	**No. birds**	58	95	109	75	125	93	555
	**No. positive**	1	6	9	4	4	6	30
	**PR (%)**	1.72	6.32	8.26	5.33	3.20	6.45	5.41
	**95% CI**	(0, 5.07)	(1.42, 11.21)	(3.09, 13.42)	(0.25, 10.42)	(0.11, 6.26)	(1.46, 11.44)	(3.52, 7.29)
**Passage migrant bird**	**No. birds**	0	1	2	2	0	3	8
	**No. positive**	0	0	0	0	0	1	1
	**PR (%)**	0	0	0	0	0	33.33	12.50
	**95% CI**	-	-	-	-	-	(0, 86.68)	(0, 35.42)

PR: prevalence, CI: confidence interval.

Table S6. Prevalence of *Plasmodium* spp. according to seasons

Year	2017	2018	2019	2020	2021	2022	Total
**Seasons**							

**Spring (March ~ May)**	**No. birds**	25	36	48	42	72	27	250
	**No. positive**	0	0	2	5	5	1	13
	**PR (%)**	0	0	4.17	11.90	6.94	3.70	5.20
	**95% CI**	-	-	(0, 9.82)	(2.11, 21.70)	(1.07, 12.82)	(0, 10.83)	(2.45, 7.95)
**Summer (June ~ August)**	**No. birds**	31	63	60	37	73	65	329
	**No. positive**	1	3	4	0	1	3	12
	**PR (%)**	3.23	4.76	6.67	0	1.37	4.62	3.65
	**95% CI**	(0, 9.45)	(0, 10.02)	(0.35, 12.98)	-	(0, 4.04)	(0, 9.72)	(1.62, 5.67)
**Autumn (September ~ November)**	**No. birds**	33	50	51	36	39	30	238
	**No. positive**	4	3	9	3	2	2	23
	**PR (%)**	12.12	6.00	17.65	8.33	5.13	6.67	9.62
	**95% CI**	(0.99, 23.26)	(0, 12.58)	(7.18, 28.11)	(0, 17.36)	(0, 12.05)	(0, 15.59)	(5.88, 13.36)
**Winter (December ~ February)**	**No. birds**	27	56	34	27	41	40	225
	**No. positive**	6	8	2	2	2	7	27
	**PR (%)**	22.22	14.29	5.88	7.41	4.88	17.50	12.00
	**95% CI**	(6.54, 37.90)	(5.12, 23.45)	(0, 13.79)	(0, 17.29)	(0, 11.47)	(5.72, 29.28)	(7.75, 16.25)

PR: prevalence, CI: confidence interval.

Table S7. Information on the wild birds included in this study

Detected year	Scientific name	Sampling month	Seasonal movement
2017	*Accipiter gentilis*	1	Winter migratory bird
2017	*Buteo buteo*	1	Winter migratory bird
2017	*Ixobrychus sinensis*	8	Summer migratory bird
2017	*Ixobrychus eurhythmus*	9	Summer migratory birds
2017	*Ixobrychus eurhythmus*	9	Summer migratory bird
2017	*Accipiter gentilis*	11	Winter migratory bird
2017	*Anas platyrhynchos*	11	Winter migratory bird
2017	*Corvus frugilegus*	12	Winter migratory bird
2017	*Fulica atra*	12	Resident bird
2017	*Cygnus cygnus*	12	Winter migratory bird
2017	*Corvus frugilegus*	12	Winter migratory bird
2018	*Buteo buteo*	1	Winter migratory bird
2018	*Asio otus*	1	Winter migratory bird
2018	*Anas poecilorhyncha*	1	Resident bird
2018	*Buteo buteo*	1	Winter migratory bird
2018	*Egretta alba modesta*	2	Summer migratory bird
2018	*Asio otus*	2	Winter migratory bird
2018	*Anas platyrhynchos*	2	Winter migratory bird
2018	*Pica pica*	7	Resident bird
2018	*Ixobrychus sinensis*	8	Summer migratory bird
2018	*Microscelis amaurotis*	8	Resident bird
2018	*Bubo bubo*	10	Resident bird
2018	*Ixobrychus sinensis*	10	Summer migratory bird
2018	*Pica pica*	11	Resident bird
2018	*Fulica atra*	12	Resident bird
2019	*Asio otus*	1	Winter migratory bird

2019	*Anser fabalis*	2	Winter migratory bird
2019	*Ardea cinerea*	4	Summer migratory bird
2019	*Pica pica*	4	Resident bird
2019	*Garrulus glandarius*	6	Resident bird
2019	*Microscelis amaurotis*	8	Resident bird
2019	*Bubulcus ibis*	8	Summer migratory bird
2019	*Columba livia domestica*	8	Resident bird
2019	*Oriolus chinensis*	9	Summer migratory bird
2019	*Pica pica*	9	Resident bird
2019	*Ixobrychus eurhythmus*	10	Summer migratory bird
2019	*Scolopax rusticola*	10	Winter migratory bird
2019	*Pica pica*	10	Resident bird
2019	*Zoothera dauma*	10	Summer migratory bird
2019	*Coturnix japonica*	11	Resident bird
2019	*Fulica atra*	11	Resident bird
2019	*Pica pica*	12	Resident bird
2020	*Microscelis amaurotis*	2	Resident bird
2020	*Microscelis amaurotis*	2	Resident bird
2020	*Buteo buteo*	3	Winter migratory bird
2020	*Zoothera dauma*	3	Summer migratory bird
2020	*Cyanopica cyanus*	3	Resident bird
2020	*Zoothera dauma*	4	Summer migratory bird
2020	*Zoothera dauma*	4	Summer migratory bird
2020	*Pica pica*	9	Resident bird
2020	*Anas crecca*	11	Winter migratory bird
2020	*Anas platyrhynchos*	11	Winter migratory bird
2021	*Anas platyrhynchos*	1	Winter migratory bird
2021	*Streptopelia orientalis*	2	Resident bird
2021	*Phasianus colchicus*	4	Resident bird
2021	*Egretta alba modesta*	4	Summer migratory bird
2021	*Ninox scutulata*	4	Summer migratory bird
2021	*Ninox scutulata*	5	Summer migratory bird

2021	*Streptopelia orientalis*	5	Resident bird
2021	*Columba livia domestica*	8	Resident bird
2021	*Turdus hortulorum*	9	Summer migratory bird
2021	*Ixobrychus eurhythmus*	10	Summer migratory bird
2022	*Accipiter gentilis*	1	Winter migratory bird
2022	*Columba livia domestica*	1	Resident bird
2022	*Buteo buteo*	1	Winter migratory bird
2022	*Anas strepera*	2	Winter migratory bird
2022	*Phasianus colchicus*	2	Resident bird
2022	*Pica pica*	2	Resident bird
2022	*Buteo buteo*	3	Winter migratory bird
2022	*Corvus macrorhynchos*	7	Resident bird
2022	*Accipiter gentilis*	7	Winter migratory bird
2022	*Columba livia domestica*	8	Resident bird
2022	*Pica pica*	9	Resident bird
2022	*Pernis ptilorhynchus*	10	Passage migrant bird
2022	*Buteo buteo*	12	Winter migratory bird

Table S8. Monthly average temperatures (℃) and precipitation (mm) for each year

		**Year**	**2017**	**2018**	**2019**	**2020**	**2021**	**2022**
**Seasons**								
**Spring**	March	T	6.3	8.1	7.5	7.9	8.9	7.7
		P	24.1	110.7	38.7	28.1	109.2	89.3
	April	T	13.9	13.3	12.0	10.9	13.2	13.8
		P	65.0	133.6	79.3	40.3	76.3	60.0
	May	T	18.7	17.8	18.6	17.7	16.6	18.0
		P	29.5	123.7	55.9	104.4	143.8	5.8
**Summer**	June	T	21.8	22.2	21.3	22.8	21.7	22.4
		P	60.7	132.1	143.1	184.6	91.6	188.1
	July	T	26.4	26.8	24.8	22.7	26.0	25.9

		P	308.0	172.1	217.2	420.7	233.8	178.4
	August	T	25.4	27.3	26.2	26.6	24.8	25.3
		P	241.0	282.1	140.0	401.6	288.4	305.2
**Autumn**	September	T	20.6	20.4	21.8	20.3	21.3	21.0
		P	92.1	136.5	221.2	210.0	145.8	150.8
	October	T	15.3	13.0	15.8	14.0	15.1	14.0
		P	67.6	164.2	169.0	10.5	53.9	77.9
	November	T	6.8	8.1	8.8	8.8	8.3	16.5
		P	12.7	50.5	58.2	38.9	57.3	61.6
**Winter**	December	T	-0.2	1.1	2.8	0.7	1.9	-1.4
		P	21.9	27.6	26.3	7.9	7.2	15.2
	January	T	0.1	-2.0	0.3	2.8	-0.7	-0.8
		P	15.3	21.1	8.1	83.4	20.1	2.6
	February	T	1.6	-0.2	2.4	3.6	3.6	-0.1
		P	29.8	32.5	30.8	58.3	18.7	3.5

*: Unreported data; T: average temperature; P: average precipitation.

Table S9. Birds with positive results of *Plasmodium* spp. and their lineages

Order	Scientific name	Detected year	Lineage	Rescued region	Genebank accession
**Accipitriformes**	*Accipiter gentilis*	2017	BT7	Urban	PP500635
		2017	BT7	Urban	PP500637
		2022	MELMEL01	Farmland	PP500641
		2022	SYBOR02	Urban	PP500644
	Buteo buteo	2017	BT7	Urban	PP500636
		2018	SW5	Forest	PP500638
		2018	BT7	Forest	PP500639
		2020	MELMEL01	Farmland	PP500640
		2022	CXPER01	Urban	PP500642
		2022	TURDUS1	Farmland	PP500643
		2022	SYBOR02	Urban	PP500646

	Pernis ptilorhynchus	2022	SW2	Urban	PP500645
**Anseriformes**	*Anas crecca*	2020	SW2	Urban	PP500652
	Anas platyrhynchos	2017	SW5	Urban	PP500647
		2018	SW5	Farmland	PP500650
		2020	SW2	Urban	PP500653
		2021	EMSPO06	Urban	PP500654
	Anas strepera	2022	SW5	Water	PP500655
	Anas poecilorhyncha	2018	SW5	Urban	PP500649
	Anser fabalis	2019	SYBOR02	Water	PP500651
	Cygnus cygnus	2017	SW5	Farmland	PP500648
**Charadriiformes**	*Scolopax rusticola*	2019	SW5	Urban	PP500656
**Columbiformes**	*Columba livia domestica*	2019	SERCAN01	Urban	PP500657
		2021	ZOSSEN01	Urban	PP500660
		2022	FANTAIL01	Urban	PP500661
		2022	SGS1	Urban	PP500662
	Streptopelia orientalis	2021	SW2	Urban	*PP500658*
		2021	PENPAN05	Urban	*PP500659*
**Galliformes**	*Coturnix japonica*	2019	SW5	Urban	PP500663
	Phasianus colchicus	2021	MELMEL01	Urban	PP500664
		2022	CXPER01	Urban	PP500664
**Gruiformes**	*Fulica atra*	2017	SW5	Urban	*PP500666*
		2018	SW2	Urban	*PP500667*
		2019	BT7	Urban	*PP500668*
**Passeriformes**	*Corvus frugilegus*	2017	PADOM02	Urban	*PP500669*
		2017	DONANA02	Urban	*PP500670*
	Corvus macrorhynchos	2022	SYCON02	Urban	PP500691
	Cyanopica cyanus	2020	SGS1	Urban	PP500685
	Garrulus glandarius	2019	SGS1	Urban	PP500675
	Pica pica	2018	SGS1	Urban	PP500671
		2018	SGS1	Urban	PP500673
		2019	RBQ03	Urban	PP500674
		2019	YWT4	Urban	PP500678

		2019	YWT4	Urban	PP500679
		2019	PAHIS07	Urban	PP500681
		2020	SGS1	Urban	PP500688
		2022	SGS1	Urban	PP500690
		2022	SGS1	Urban	PP500692
	Oriolus chinensis	2019	SGS1	Urban	PP500677
	Microscelis amaurotis	2018	SGS1	Urban	PP500672
		2019	SGS1	Urban	PP500676
		2020	PORUF03	Urban	PP500682
		2020	ALERUF05	Urban	PP500683
	Zoothera dauma	2019	TURPAL01	Urban	PP500680
		2020	TURPAL01	Urban	PP500684
		2020	SYAT05	Urban	PP500686
		2020	TURPAL05	Urban	PP500687
	Turdus hortulorum	2021	SYAT05	Urban	PP500689
**Pelecaniformes**	*Ardea cinerea*	2019	ACCBAD01	Urban	PP500699
	Bubulcus ibis	2019	PICAN01	Farmland	PP500700
	Egretta alba modesta	2018	CXPIP24	Water	PP500696
		2021	NYCNYC02	Farmland	PP500702
	Ixobrychus eurhythmus	2017	DONANA02	Urban	PP500694
		2017	SW5	Urban	PP500695
		2019	SW5	Urban	PP500701
		2021	DONANA02	Urban	PP500703
	Ixobrychus sinensis	2017	DONANA02	Urban	PP500693
		2018	CXINA01	Urban	PP500697
		2018	CXINA01	Urban	PP500698
**Strigiformes**	*Asio otus*	2018	SW5	Urban	PP500704
		2018	SW5	Farmland	PP500705
		2019	SW5	Water	PP500707
	Bubo bubo	2018	CXINA01	Forest	PP500706
	Ninox scutulata	2021	NISCU1	Urban	PP500708
		2021	FANTAIL01	Urban	PP500709
